# Research of Cu-Doped Hydroxyapatite Microbeads Fabricated by Pneumatic Extrusion Printing

**DOI:** 10.3390/ma12111769

**Published:** 2019-05-31

**Authors:** Wenchao Chi, Jiawei Zou, Fanrong Ai, Yanjun Lin, Wenchao Li, Chuanliang Cao, Kang Yang, Kui Zhou

**Affiliations:** 1 School of Mechatronics Engineering, Nanchang University, Nanchang 330031, China; wcchi@email.ncu.edu.cn (W.C.); JiaweiZOU@email.ncu.edu.cn (J.Z.); aifanrong@ncu.edu.cn (F.A.); yjlin@email.ncu.edu.cn (Y.L.); Chadli@ncu.edu.cn (W.L.); cl_cao@ncu.edu.cn (C.C.); 2 Key Laboratory of Lightweight and high strength structural materials of Jiangxi Province, Nanchang University, Nanchang 330031, China; 3 College of Pharmacy, Nanchang University, Nanchang 330031, China; yangkang@email.ncu.edu.cn; 4 Engineering Science and Mechanics Department, Penn State University, University Park, PA 16802, USA

**Keywords:** hydroxyapatite, microbeads, copper, biological performance

## Abstract

Copper is an indispensable micronutrient in human health, which has important effects on the promotion of angiogenesis and thus contributes to bone formation and antimicrobial activity. We used ion exchange and pneumatic printing methods to prepare hydroxyapatite (HA) microspheres with different copper content. The microspheres were characterized by scanning electron microscope (SEM), X-ray diffractometry (XRD) and X-Ray photoelectron spectroscopy (XPS). Considering the resistance of hydroxyapatite to biodegradation in vivo, the degradation rate of microspheres in modified simulated body fluids was studied. In addition, cell proliferation and antibacterial experiments were carried out to study the biological properties of microspheres. HA-1.5MCu microbeads treated by 1.5 mol/L CuSO_4_ curing solution have good performance on degradation, antibacterial properties and cell survival rate on day 7. The results showed that HA-1.5MCu microbeads may be used as a good repair material for bone defects.

## 1. Introduction

Hydroxyapatite (Ca_10_(PO_4_)_6_(OH)_2_,HA) is the main inorganic component of the human skeleton. HA is widely used in hard tissue repair and replacement due to its good biocompatibility and bioactivity. However, the main component of human bone mineral is not pure HA, but the typical calcium-deficient HA, whose expression is M_10_(ZO_4_)_6_X_2_. M is an alternative position that can be replaced by cations (such as Mg^2+^, Zn^2+^, Sr^2+^, Cu^2+^) [1]. Hydroxyapatite ceramic microspheres play an important role in cancer therapy, antibacterial therapy, and other biomedical applications [2,3]. In addition, ceramic microspheres can be widely used in stomatology and bone defect filling in other parts of human bones [4].

Moreover, copper-doped microspheres have their own unique functions. Copper, as the second essential trace element in human body, plays an important role in the human metabolism [5]. Copper can promote the growth of vascular endothelial cells and facilitate angiogenesis [6]. At the same time, copper can promote the formation of collagen, thereby maintaining the normal composition and structure of bones and inhibiting osteoporosis [7]. In addition, copper has good antibacterial activity against both gram-positive and negative bacteria [8]. Therefore, the addition of copper to the synthetic HA microsphere is expected to improve its biological properties and give it an antibacterial function. Currently, copper-doped HA is the main preparation method for copper-doped microspheres, while the main synthesis methods for copper-doped HA include chemical coprecipitation [9], high-temperature solid-phase synthesis [10], and ion exchange [11]. The process of chemical coprecipitation is simple, but the product has poor crystallinity and low purity [9]. High temperature solid phase synthesis requires high reaction temperature, and the product is easy to agglomerate. However, the above methods have the disadvantages of complex and unstable processes, and no one has studied how to turn doping copper into commercial HA powders. Copper alginate colloidal spheres can be produced by dropping alginate solution into aqueous solution containing copper ions, and then the copper-doped microspheres can be produced by sintering.

The copper-doped hydroxyapatite ceramic microspheres were obtained by the pneumatic printing method. The sintering process was determined by thermogravimetric analysis (TGA). The effects of different doping amounts on the physicochemical and biological properties of hydroxyapatite ceramic microspheres were investigated. The physical properties of hydroxyapatite ceramic microspheres were characterized by X-ray diffractometry (XRD), X-Ray photoelectron spectroscopy (XPS), and scanning electron microscope (SEM). The effects of different doping amounts on the water absorption and degradation rate were also studied. The biological properties of Cu-doped microspheres were characterized by antibacterial and cell experiments.

## 2. Materials and Methods

### 2.1. Materials

Nano HA powder (needle like particle, 60 nm width and 150 nm length) was supplied by Emperor Nano Material, China. Sodium alginate (SA) used in the experiment was purchased from Aladdin Industrial Inc. CuSO_4_·5H_2_O (analytical grade) were obtained from Sinopharm Chemical Reagent Co. Ltd.

### 2.2. Preparation of Cu-doped HA Microbeads via Pneumatic Extrusion Printing

**Figure 1:**
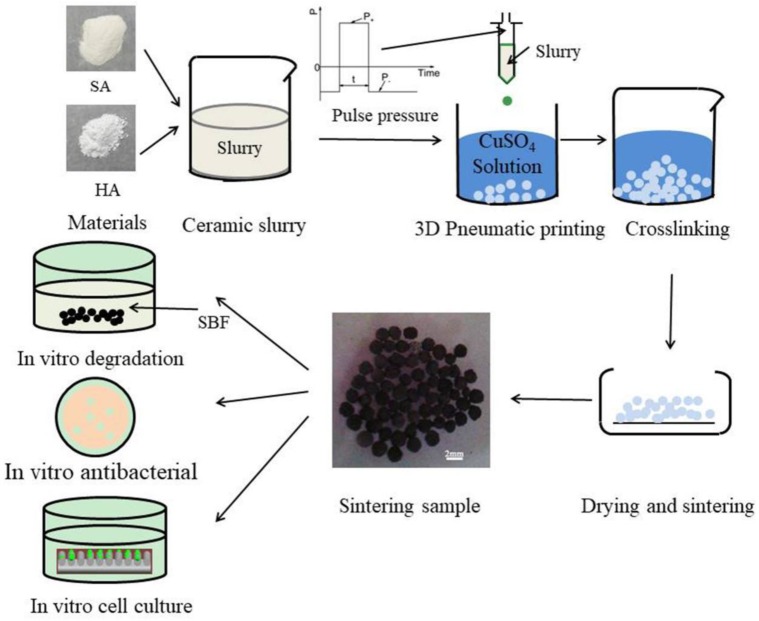
Schematic representation of the preparation protocol for the Cu-doped hydroxyapatite (HA) macrobeads.

Cu-doped HA microbeads were fabricated through the preparation protocols illustrated in Figure 1. First, the HA suspension was prepared. In a typical experiment, 1 g of sodium alginate and 10 g HA powder were added to 100 g distilled water with continuous stirring by juice extractor to obtain homogeneous HA suspension. After the suspension was prepared, it was printed into CuSO_4_ solutions by a homemade pneumatic extrusion printing to obtain microbeads. A cone angle needle with an inner diameter of 0.6 mm was used. The printing parameters for positive pressure, negative pressure, dwell time and collecting distance were 30 kPa, 7 kPa, 10 ms, 60 mm, respectively. The obtained HA microbeads were crosslinked in CuSO_4_ solutions (0.1 mol/L, 0.5 mol/L, 1.0 mol/L and 1.5 mol/L) for 24 h and dried at room temperature, named as HA-0.1MCu, HA-0.5MCu, HA-1MCu and HA-1.5MCu microbeads respectively. Prior to the sintering step, the TGA of HA-0.5MCu microbeads was conducted from 30 °C to 900 °C at a heating rate of 10 °C/min, using a PerkinElmer TGA-4000 apparatus (Perkin-Elmer, Wellesley, USA). The optimal sintering parameters of the dried microbeads were defined as: (1) pre-sintered at 400 °C for 1 h; and (2) Sintered at 1200 °C for 2 h using a commercial oven (SX2-10-13, Jiangsu qianjin furnace industry, China). 

### 2.3. Characterization of Cu-Doped HA Microbeads

#### 2.3.1. Physico-Chemical Characterization

The morphologies of the sintered Cu-doped HA microbeads were characterized by SEM (SIGMA300, Zeiss, Jena, Germany) at an operating voltage of 3 kV after sputtered-coated with gold. The phases of microbeads were characterized by X-ray diffractometry (D8 ADVANCE, BRUKER, Karlsruhe, Germany), using 40 kV, 40 mA, Cu Kα X-rays. The XRD test conditions were as follows: scan rate 4 °/min, step 0.02°, scan range 20°–60° and X-ray wavelength λ = 0.1542 nm. X-Ray photoelectron spectroscopy (XPS ESCALAB250Xi, ThermoFisher Scientific, Waltham, USA) of the samples was undertaken out using a ThermoFisher Scientific ESCALAB250XiX spectrometer to investigate the successful doping of HA with Cu. Al-Kα irradiation was used for the target (1486.6 eV). The beam spot size was 650 µm. Survey scans were recorded with a 0.1 eV step and 30 eV pass energy. The water absorption tests were carried out by soaking samples in distilled water for 24 h and then tapered with a soft paper towel to remove excess water from the specimen surface [12]. The water absorption (WA %) was calculated according to the following equation:

(1) $$\mathrm{WA}\left(\%\right)=\frac{W_{1}-W_{2}}{W_{1}}\times 100$$

where W_1_ and W_2_ are weights of the sintered Cu-doped HA microbeads before and after immersing in distilled water of 24 h, respectively. The weight of Cu-doped HA microbeads were characterized by an electronic analytical balance ( JJ224BF Changshu Shuangjie test instrument factory China). The accuracy of the analytical balance was 0.0001 g. Four parallel samples (~0.9 g microbeads for each sample) were used for the water absorption test. Since HA degrades very slowly in a neutral environment [13], the acidic simulated body fluid (SBF, pH = 3) was used in this study to accelerate the degradation process [14]. Cu-doped HA microbeads were immersed into SBF solution with solid/liquid ratio of 0.1 g/mL. The SBF solution was changed every day. The microbeads were removed from the SBF at day 0, 1, 3, 5, 7, 10, 14 and 17, dried in a drying oven. The percentage of weight loss was calculated as follows:

(2) $$\mathrm{Weight}\;\mathrm{Loss}\left(\%\right)=\frac{M_{0}-M}{M_{0}}\times 100$$

where *M*_0_ is the starting dry weight and *M* is the dry weight at day *t*. Weight loss experiments were performed in three trials. Electronic analytical balance (JJ224BF Changshu Shuangjie test instrument factory China, accuracy is 0.0001 g) was used to measure the weight of Cu-doped HA microbeads.

#### 2.3.2. Antimicrobial Analysis

S. aureus and E. coli are clinically common pathogenic bacteria. The antibacterial activity of the Cu-doped HA microbeads against S. aureus (ATCC 25923) and E. coli (ATCC 25922) [15] was studied using the mean colony-forming units method. 0.025 g Cu-doped HA microbeads were put into a flask containing 2 mL bacterial suspension and incubated at 37 °C for 24 h. The samples were taken out and 100 μL of 10^−6^ dilutions were plated out on TSA plates and incubated overnight at 37 °C. The colonies were counted on the following day. The number of viable microorganism colonies was determined using the method of plate counting and the antibacterial ratio was calculated as follows:

(3) $$\mathrm{Antibacterial}\;\mathrm{ratio}\left(\%\right)=\frac{N_{1}-N_{2}}{N_{1}}$$

where *N*_1_ is the average value of survived bacteria incubated with control groups, and *N*_2_ is the number of survived bacteria incubated with Cu-doped HA microbeads. The experiments were carried out in triplicate to confirm reproducibility.

#### 2.3.3. In Vitro Cell Culture Evaluation

To prepare Cu-doped HA microbeads extracts, 0.05 g of microbeads were placed into 2.5 mL of complete culture medium. The medium was then incubated at 37 °C (in a humidified atmosphere of 5% CO_2_) for 24 h. Then, the extract was collected from the medium. Such obtained extracts were subjected to evaluation of cell proliferation. Bone marrow mesenchymal stem cells (BMMSCs) [16], harvested from one month old male SD rats, were used to assess the cytocompatibility of the microbeads. BMMSCs were co-cultured with 10 μL Cu-doped HA microbeads extracts at a density of 2 × 10^3^ cells/well in 96-well plate. The Cell Counting Kit-8 assay (CCK8; Dojindo Molecular Technologies Inc., Japan) were tested after day 1, day 3 and day 7. The relative increase in the rate of cells in each experimental group was calculated by the mean value of the absorbance of the blank control group being 100% cell proliferation rate. Three parallel samples were used for the cell culture experiment.

#### 2.3.4. Statistical Analyses

The data was expressed as mean ± standard deviation (SD). Statistical analysis was performed on Statistical Product and Service Solutions software (SPSS 17.0, IBM, USA) using one-way ANOVA, followed by the Tukey post hoc test for multiple groups comparison. A p value <0.05 was considered to be statistically significant.

## 3. Results and Discussion

**Figure 2:**
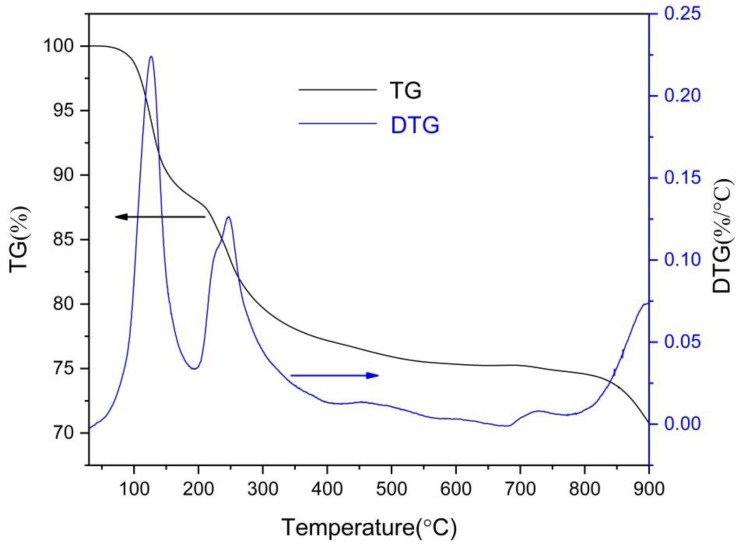
Thermogravimetric analysis (TGA) curves of HA-0.5MCu macrobeads.

TGA experiments were used to validate the loss of mass during the sintering. The TGA curves shown in Figure 2 exhibit three stages of weight loss up to 900 °C. The first drop in the weight loss around 100 °C is due to the loss of water in the scaffold. Then the second weight loss that occurs between 200 °C and 400 °C can be associated with the pyrolysis of salt alginate. The last weight loss above 850 °C is due to the decomposition of the pyrolysis product of salt alginate and the dehydroxylation behavior of HA. Thus, a staged sintering process was used in this research.

**Figure 3:**
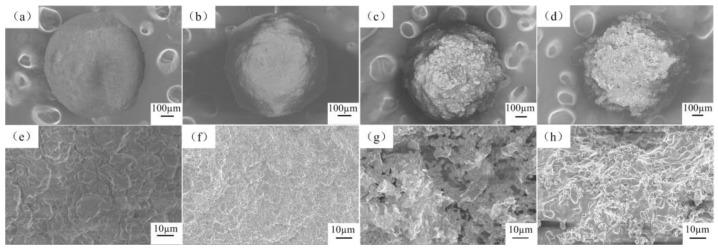
Scanning electron micrograph of Cu-doped HA macrobeads: (**a**,**e**) HA-0.1MCu, (**b**,**f**) HA-0.5MCu, (**c**,**g**) HA-1.0MCu, (**d**,**h**) HA-1.5MCu.

The SEM images of the surface cross-section of the sintered microbeads are shown in Figure 3. The Cu-doped microspheres with HA-0.1MCu have good microbeads shape on a macroscopic and compact surface structure on microscopic. With the increase of curing solution concentration, the surface of the copper-doped microbeads becomes rougher at the macro level. In particular, HA-1.0MCu and HA-1.5MCu microbeads show obvious uneven structure at the micro level, but maintain a good microbeads shape on the whole.

**Figure 4:**
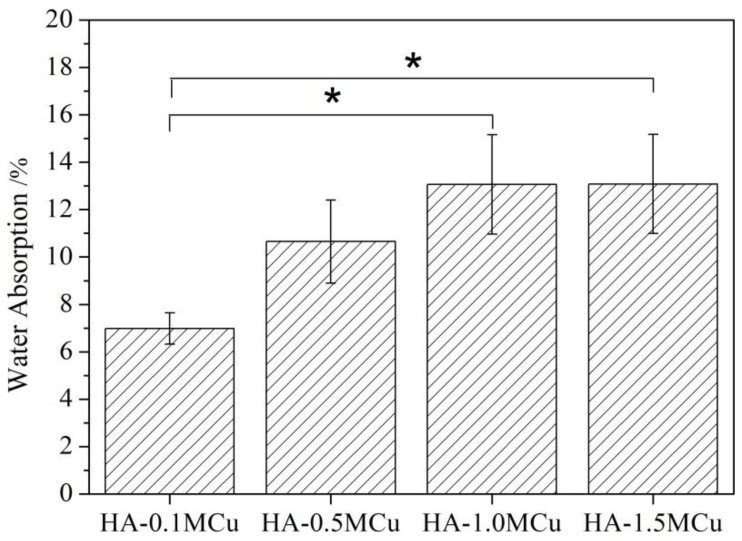
Water absorption of Cu-doped HA macrobeads after sintering. Asterisks (*) denote. significant differences (* *p* < 0.05).

In order to further explore the microstructure of Cu doped microbeads, the water absorption rate of Cu doped microbeads was studied by liquid replacement method [17] at room temperature. The water absorption rate images are shown in Figure 4. The results showed that the water absorption rate of HA-1.0MCu and HA-1.5MCu microspheres was significantly higher than that of HA-0.1MCu microspheres. Combining with SEM results, it may be due to the HA-1.0MCu and HA-1.5MCu microbeads having a rougher surface structure. 

**Figure 5:**
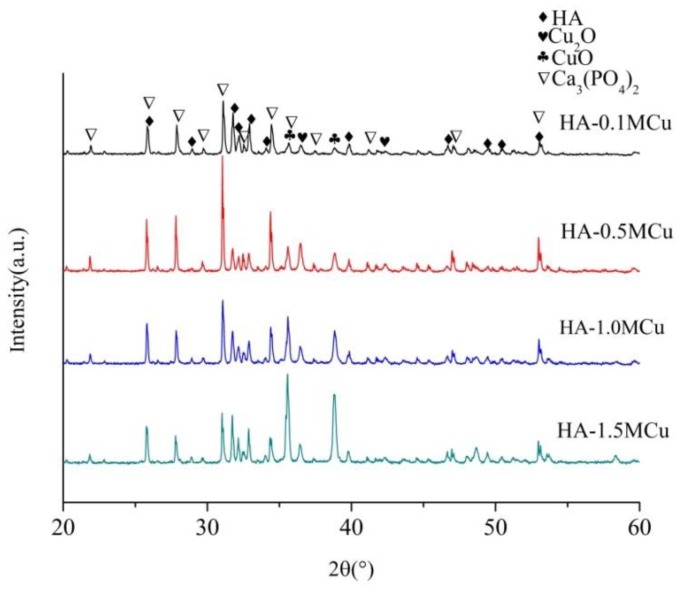
XRD patterns of Cu-doped HA macrobeads after sintering.

The XRD patterns of the Cu-doped microbeads after sintered at 1200 °C are shown in Figure 5. Similar to the XRD lines of Cu doped microbeads, all of the Cu-doped microbeads are composed of hydroxyapatite (JCPDS-PDF: 09-0432) [12], tricalcium phosphate [18] (JCPDS-PDF: 70-2065), copper oxide (JCPDS-PDF: 80-0076) and cuprous oxide [19] (JCPDS-PDF: 78-2076). Hydroxyapatite will convert into tricalcium phosphate when the temperature is above 1300 °C [20]. But in this experiment, tricalcium phosphate was produced in 1200 °C. The reason may be that the incorporation of copper leads to lower temperature of hydroxyapatite converted into tricalcium phosphate [21]. A similar phenomenon can be seen in Silva's study [22]. Zn-alginate–hydroxyapatite composite spheres were produced in a solution rich in zinc ions. During sintering, zinc ions entered the hydroxyapatite structure and induced its decomposition to β-TCP. The pyrolysis of copper alginate produces copper oxide and cuprous oxide.

**Figure 6:**
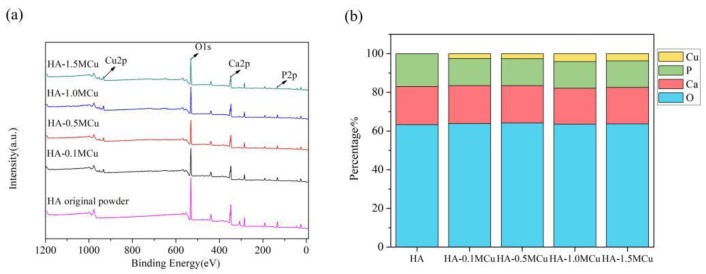
XPS patterns of Cu-doped HA macrobeads after sintering (**a**) and atomic ratio of Ca, P, O and Cu (**b**).

The XPS patterns of Cu-doped HA microbeads after sintering at 1200 °C are shown in Figure 6. The sintered microspheres mainly contained Ca, P, O and Cu elements. The atomic ratios of Ca, P, O and Cu are shown in the Figure. With the increase of the concentration of curing solution, the amount of copper in the microspheres increased. When the concentration was low (0.1 mol/L, 0.5 mol/L, 1.0 mol/L), the content of copper in the microspheres increased rapidly, while when the concentration reached 1.0 mol/L, the increase of copper content slowed down. Therefore, the doping amount of copper can be adjusted by adjusting the concentration of the curing solution.

**Figure 7:**
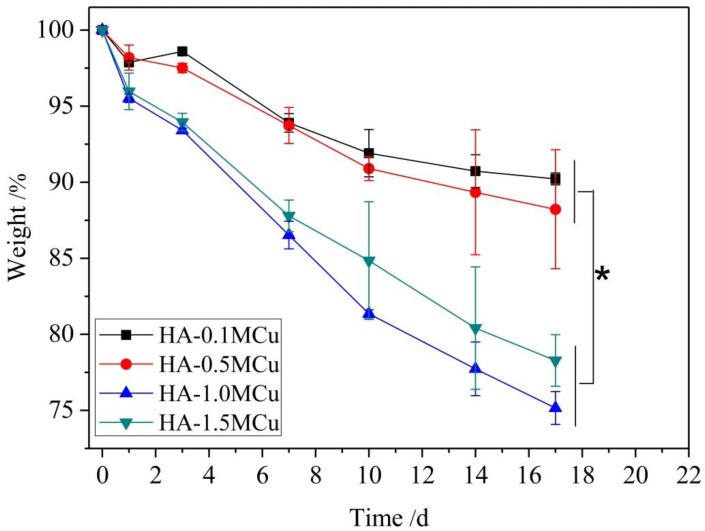
Weight loss rate of sintered Cu-doped HA macrobeads in acidic SBF (pH = 3). (* *p* < 0.05).

The degradation properties of Cu-doped beads were determined. According to the Figure 7, the degradation rate of HA-1.0MCu and HA-1.5MCu copper-doped microspheres was significantly faster than that of HA-0.1MCu and HA-0.5MCu copper-doped microspheres. The reason is that HA-1.0MCu and HA-1.5MCu copper-doped microspheres have a relatively rougher surface structure. As a result, the contact area between the microspheres and SBF (pH = 3) during the degradation process is larger, so the degradation rate of HA-1.0MCu and HA-1.5MCu copper-doped microspheres is faster. Nevertheless, the degradation process of all microspheres showed a similar trend, that is, the degradation degree increased with the increase of degradation time.

**Figure 8:**
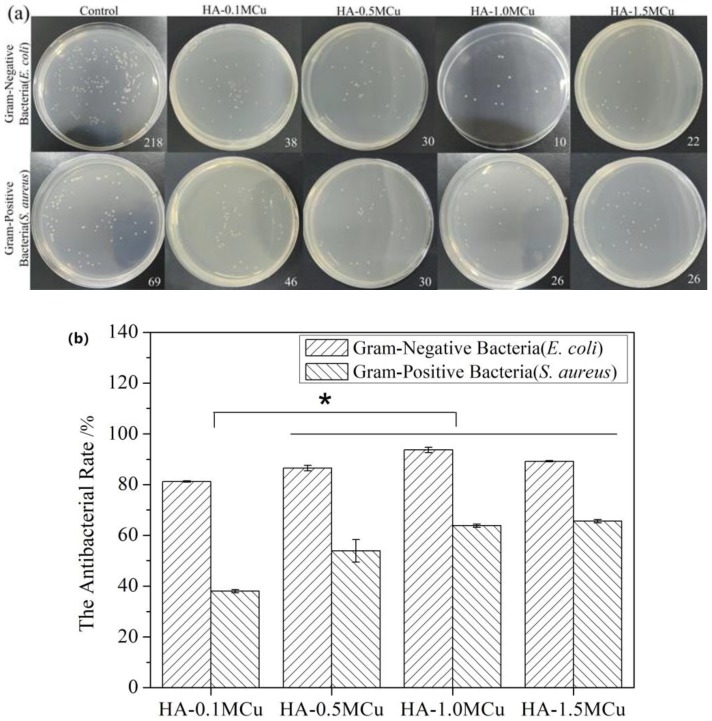
Antibacterial rate against E. coli and S. aureus. (* *p* < 0.05). (**a**) Plate counting photographs; (**b**) quantitative antibacterial results.

The effect of copper ions in copper-doped microspheres on the antimicrobial activity was determined by plate method using E. coli and S. aureus strains. The possible mechanism of action of copper as an antibacterial agent in microspheres could be due to the interaction of copper ions with the outer membrane of the bacteria and thereby causing structural damage and finally bacteria death. According to the Figure 8, we can see that the antibacterial activity of Cu-doped microspheres against gram-negative bacteria was higher than that of gram-positive bacteria [23]. However, the antimicrobial activity of HA-1.0MCu and HA-1.5MCu copper-doped microspheres was significantly higher than that of HA-0.1MCu copper-doped microspheres. The reason is that copper has good antibacterial performance [24], and compared with HA-0.1MCu copper doped microspheres, HA-1.0MCu and HA-1.5MCu copper doped microspheres have higher Cu content and rougher surface structure. As a result, the copper-doped microspheres of HA-1.0MCu and HA-1.5MCu lead to more Cu elements diffusing into the leaching solution, which improves the antimicrobial performance of the microspheres. Thus, the antimicrobial effect can be improved by tailoring the parameters of the concentration of the curing solution.

**Figure 9:**
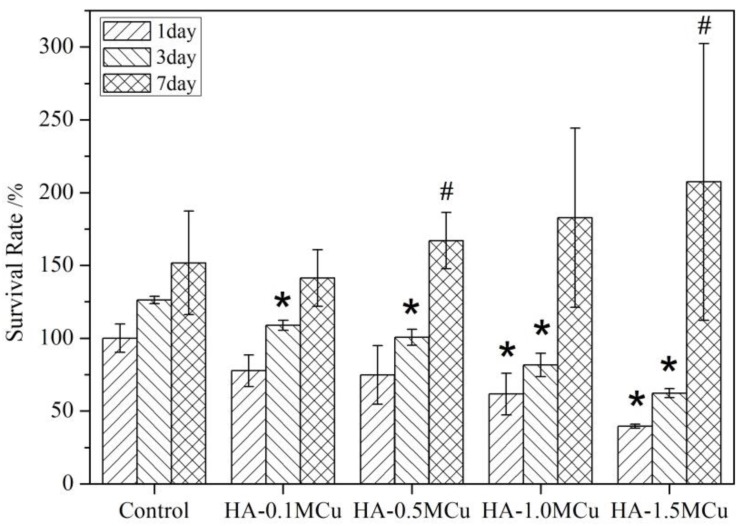
Cell survival rate. (* *p* < 0.05, compared to control group; # *p* < 0.05, compared to day 1).

The cell survival rates are shown in Figure 9. It can be seen that the cell survival rate of the HA-1.0MCu and HA-1.5MCu microspheres on day 1 was significantly lower than that of the control group on day 1. The cell survival rate of the HA-0.1MCu, HA-0.5MCu, HA-1.0MCu and HA-1.5MCu microspheres on day 3 was significantly lower than that of the control group on day 3. There was no significant difference between the cell survival rate on day 7 and that of the control group on day 7. This indicates that the presence of Cu ion has obvious cytotoxicity at the early stage of the experiment. The higher the concentration of copper ions, the greater the cytotoxicity. However, the cell survival rate of HA-0.5MCu on day 7 was significantly higher than that of HA-0.5MCu on day 1, and that of HA-1.5MCu on day 7 was significantly higher than that of HA-1.5MCu on day 1, indicating that the microspheres of HA-0.5MCu and HA-1.5MCu promoted the cell survival in the later stage of the experiment. The reason may be that copper ions have two effects on cell proliferation activity. On the one hand, copper ion incorporation positively promoted the expression of hypoxia-inducible factor 1-alpha in BMMSCs and promoted the proliferation activity of cells [25]. On the other hand, when the concentration of copper ions is high, it has an inhibitory effect on cell proliferation activity [26]. On the first and third days, the cell survival rate decreased with an increase in the concentration of copper ions, which may be due to the toxicity of copper ion. By day 7, the burst release period of copper ions had passed and the cells had adapted to the environment. Thus the positive effect of copper ions on cell proliferation activity began to emerge, which directly resulted in the cell survival rate of the experimental group began to increase, and with the increase of copper content in the microspheres, the cell survival rate also increased. The promoting effect of HA-1.0MCu and HA-1.5MCu microbeads on BMMSCs may help us to use these microbeads as a good repair material for bone defects.

In general, microspheres with higher copper content (HA-1.5MCu) have higher antibacterial activity, which may play a good role in the prevention of wound infection caused by traumatic wounds or surgical implantation, but the specific antibacterial prevention of wounds still needs further research.

## 4. Conclusions

In this paper, a facile method for preparing HA ceramic microbeads is established. Compared with microbeads with low copper doping amount, microbeads with high copper doping amount have rougher surface structure, which are of great significance in biological properties, including biodegradation, proliferation and antibacterial. Differences in surface structure also significantly affect biodegradation, while changes in microstructure have little effect on antimicrobial activity. In addition, the cell survival rate performance of HA-1.5MCu microbeads on day 7 is good. Considering the good degradation, antibacterial performance and cell survival rate on day 7 of HA-1.5MCu microbeads, 1.5 mol/L CuSO_4_ as the curing solution may be the best choice for the scaffold material with high biological activity. The Cu-doped hydroxyapatite microbeads produced by a pneumatic printing method is cost-effective and has good antibacterial performance and cell compatibility. Hence, it could be used as an effective filling material in repairing bone and tooth defects.
